# Scaffold-Mediated Developmental Effects on Human Induced Pluripotent Stem Cell-Derived Cardiomyocytes Are Preserved After External Support Removal

**DOI:** 10.3389/fcell.2021.591754

**Published:** 2021-02-15

**Authors:** Jun Li, Jong-Kook Lee, Keiko Miwa, Yuki Kuramoto, Kiyoshi Masuyama, Hideki Yasutake, Satoki Tomoyama, Hiroyuki Nakanishi, Yasushi Sakata

**Affiliations:** ^1^Department of Cardiovascular Medicine, Osaka University Graduate School of Medicine, Suita, Japan; ^2^Department of Cardiovascular Regenerative Medicine, Osaka University Graduate School of Medicine, Suita, Japan; ^3^Department of Medical Laboratory Science, Faculty of Health Sciences, Hokkaido University, Sapporo, Japan

**Keywords:** hiPS-CMs, development, tissue engineering, geometric induction, alignment, maturation

## Abstract

Human induced pluripotent stem (hiPS) cells have been used as a cell source for regenerative therapy and disease modeling. The purity of hiPS-cardiomyocytes (hiPS-CMs) has markedly improved with advancements in cell culture and differentiation protocols. However, the morphological features and molecular properties of the relatively immature cells are still unclear, which has hampered their clinical application. The aim of the present study was to investigate the extent to which topographic substrates actively influence hiPS-CMs. hiPS-CMs were seeded on randomized oriented fiber substrate (random), anisotropic aligned fiber substrate (align), and flat non-scaffold substrate (flat). After culturing for one week, the hiPS-CMs on the aligned patterns showed more mature-like properties, including elongated rod shape, shorter duration of action potential, accelerated conduction velocity, and elevated cardiac gene expression. Subsequently, to determine whether this development was irreversible or was altered after withdrawal of the structural support, the hiPS-CMs were harvested from the three different patterns and reseeded on the non-scaffold (flat) pattern. After culturing for one more week, the improvements in morphological and functional properties diminished, although hiPS-CMs pre-cultured on the aligned pattern retained the molecular features of development, which were even more significant as compared to that observed during the pre-culture stage. Our results suggested that the anisotropic fiber substrate can induce the formation of geometrical mimic-oriented heart tissue in a short time. Although the morphological and electrophysiological properties of hiPS-CMs obtained via facilitated maturation somehow rely on the existence of an exterior scaffold, the molecular developmental features were preserved even in the absence of the external support, which might persist throughout hiPS-CM development.

## Introduction

Cardiovascular diseases are one of the main causes of death worldwide (Gersh et al., 2010). The treatment options for heart failure, the end stage of most cardiovascular diseases, are limited, as treatment validity among patients is mixed owing to individual differences. Recently, prominent advancements have been made toward cardiac regenerative therapy (Zhang, 2015; Ichimura and Shiba, 2017). Furthermore, pluripotent stem cell-derived cardiomyocytes (PSC-CMs) are being considered as promising candidates for understanding cardiac physiological development (Kolanowski et al., 2017; Scuderi and Butcher, 2017) and pathological progression (Yazawa et al., 2011; Sun et al., 2012; Chun et al., 2015; Sakai et al., 2018). However, the characteristics of immature CMs, from morphological features to molecular properties, are still not clear, which has hampered their clinical application (Veerman et al., 2015; Yoshida and Yamanaka, 2017). To overcome these fundamental limitations, various strategies for facilitating PSC-CM maturation, such as topographic induction (Kim et al., 2012), mechanical or electrical stimulation (Ruan et al., 2016), supplementation with biochemical factors (Engels et al., 2014; Yang et al., 2014), and genetic manipulation (Anderson et al., 2007) have been used. While these methods improved CM maturation to a certain extent, disparities with native cardiac tissue still exist (Veerman et al., 2015). For instance, the native heart tissue consists of well-aligned cardiac laminar layers (Streeter et al., 1969; LeGrice et al., 1995), which guarantee high efficiency of contractility as well as electrical conductivity (Hooks et al., 2002). Several reports have suggested that well-aligned cardiac tissue can be successfully obtained by culturing PSC-CMs on anisotropic pattern, although its effects on promotion of PSC-CM maturation are still ambiguous (Pilarczyk et al., 2016).

In this study, we confirmed the beneficial effects of aligned topography on hiPS-CM maturation. Furthermore, we investigated if the benefits remained in the mature cells even after removal of the topographic stimuli. The functional maturation of hiPS-CMs relies on extracellular exterior structural support, but the positive effects due to the support may persist throughout hiPS-CM development. Determining the hiPS-cardiac cellular “memory” for topographic effects is crucial for their application in regenerative medicine in a clinical setting.

## Materials and Methods

### hiPSC Culture and Cardiac Differentiation

hiPS cells, 201B7 (RIKEN Bioresource Center, Tsukuba, Japan), were maintained in StemFit AK02 medium (AK02N; Ajinomoto). Cardiac differentiation was induced using the cardiomyocyte differentiation kit (A2921201; Gibco). The culture medium was replaced first with medium A and then with medium B after every 2 days, and then the cells were finally maintained in cardiomyocyte maintenance medium for 7 days. On day 14, the culture medium was replaced by non-glucose Roswell Park Memorial Institute (RPMI) medium (11879-020; Gibco) containing 5 mM lactic acid (128-00056; Wako) and 0.1% bovine serum albumin (BSA, 037-23372; Wako) (Tohyama et al., 2013) and incubated for 8 days. Then, the cultures were maintained in cardiomyocyte maintenance medium until further analysis. All cells were maintained at 37°C in a 5% CO2-humidified incubator.

### hiPS-CMs Culture on Different Patterns

On post-differentiation day 30, hiPS-CMs were dissociated into single cells using 0.25% trypsin-ethylene diamine tetraacetic acid (25200072; Gibco) and seeded on 0.1% gelatin pre-coated flat bottom 96-well plate (PerkinElmer, 6005550)/random nanofiber substrate (NanoECM, 9601; Funakoshi)/aligned nanofiber substrate (NanoAligned, 9602; Funakoshi) either at the density of 3.125 × 10^5^ cells/cm^2^ for functional analysis or 3.125 × 10^4^ cells/cm^2^ for morphological study. The cultures were maintained in M199 (12350-039; Gibco) with 10% fetal bovine serum (FBS) for 7 days. hiPS-CMs from the same patch were seeded on 0.1% gelatin flat bottom pre-coated 24-well plate (MS-80240; Sumilon)/random nanofiber substrate (NanoECM, 2401; Funakoshi)/aligned nanofiber substrate (NanoAligned, 2402; Funakoshi) at the density of 3.125 × 10^5^ cells/cm^2^. After culturing for 7 days, the cells were harvested for quantitative reverse transcription-polymerase chain reaction (qRT-PCR). The plates were all xeno-free, and the nanofibers were made of polycaprolactone; the diameter of the nanofibers were approximately 700 nm.

### De-Scaffold Culture of hiPS-CMs

hiPS-CMs pre-cultured on the different patterns for 7 days were harvested and re-plated on non-scaffold (flat bottom) 96-well or 24-well plates and cultured for another 7 days. Thereafter, the cultures in the 96-well plates were used for morphological and functional studies, and those in the 24-well plates were harvested for molecular analysis.

### Immunofluorescence Staining and Imaging Analyses

Cells were fixed in 4% paraformaldehyde (PFA) for 15 min at 25°C. After permeabilization in 0.1% Triton X-100 for 10 min, the samples were blocked with 2% BSA for 1 h at RT. The primary antibodies, anti-cardiac troponin-T (1:200, catalog number MS-295-P1; Lab Vision) and anti-connexin-43 (1:200, catalog number 710700; Abcam), diluted in the blocking solution were applied to the sample and incubated overnight at 4°C. After washing thrice with phosphate-buffered saline (PBS) for 5 min each, appropriate secondary antibodies (Alexa Fluor 488 and Alexa Fluor 647; 1:500 dilution) in 2% BSA were applied and incubated in the dark for 1 h at RT. Finally, after rinsing thrice in PBS for 5 min each, the cultures were mounted with Hoechst 33342 (1:1000, Dojindo, H342). The stained images were visualized using a high content imaging system (Operetta, PerkinElmer, Waltham, MA, United States). Cell shapes were analyzed using the Harmony analysis software (PerkinElmer).

### Contractility of Spontaneously Beating hiPS-CMs

Free labeling motion analysis of hiPS-CMs was performed using the cell motion imaging system (SI8000C, Cardio Model, Sony, Tokyo, Japan), as previously reported (Iseoka et al., 2018). Briefly, spontaneously beating hiPS-CMs were monitored at 37°C in a 5%-CO2 incubator. Images were captured using a 10 × objective at a frame rate of 150 fps for 5 s. The data were analyzed using a SI8000C analyzer software (Sony).

### Calcium Transient and Membrane Potential

For calcium transient, the cells were loaded with 5 μM Indo-1 AM (1006; Dojindo) dissolved in FluoroBrite Dulbecco’s modified Eagle’s medium (DMEM) (A1896701; Gibco) containing 0.1% pluronic F-127 (P3000MP; Thermo Fisher Scientific) and incubated at 37°C for 1 h. After washing twice with PBS, the cells were incubated in FluoroBrite DMEM for 30 min at 37°C. For measuring membrane potential, the prepared cells were treated with the FluoVolt membrane potential kit (F10488; Thermo Fisher Scientific) for 30 min at 37°C. The medium was replaced with FluoroBrite DMEM containing 2% FBS and incubated for another 30 min. The calcium transient and membrane potential were measured under pacing at 1 Hz. The imaging data were acquired and analyzed using the FDSS/uCELL system (C13299; Hamamatsu Photonics, Hamamatsu, Japan).

### Optical Mapping

Cells were prepared similar to that for membrane potential measurement and treated with the membrane potential kit (F10488; Thermo Fisher Scientific). Optical mapping was performed using the MiCAM02 imaging system (BrainVision, Tokyo, Japan) combined with MyoPacer EP (IonOptix, Westwood, MA, United States), as reported previously (Nakanishi et al., 2019). Briefly, after loading the cells, the cultures were electrically stimulated using a bipolar electrode. For aligned pattern, pacing was performed parallel or perpendicular to the direction of arranged nanofibers, using the same pacing positions for random and flat patterns. The pacing rate ranged from 0.5 Hz to 2 Hz in a gradient. The whole procedure was performed at 37°C under atmospheric conditions. Optical imaging was performed using the BV_Ana software (BrainVision, Tokyo, Japan).

### qRT-PCR

Total RNA was extracted using the RNeasy Plus mini kit (740990.250; Takara) and cDNA was synthesized using the SuperScript VILO cDNA synthesis kit (11754-250; Thermo Fisher Scientific). Next, qRT-PCR was performed using the Taqman Fast advanced master mix (4444557; Thermo Fisher Scientific) and assessed using the ViiA 7 real-time PCR system (Thermo Fisher Scientific). The expression of the target gene was normalized to that of 18S rDNA, the internal control (18s rRNA, Hs99999901_s1). The genes analyzed using the TaqMan gene expression assays were as follows: *MYH6* (Hs01101425_m1), *MYH7* (Hs01110632_m1), *MYL2* (Hs00166405_m1), *MYL7* (Hs01085598_g1), *SCN5A* (Hs00165693_m1), *GJA1* (Hs00748445_s1), *ATP2A2* (Hs00544877_m1), *CACNA1C* (Hs00167681_m1), *KCNJ2* (Hs01876357_s1), *KCNQ1* (Hs00923522_m1), and *KCNH2* (Hs04234270_g1).

### RNA-Sequencing (RNA-Seq)

Total RNA was extracted using the RNeasy Plus mini kit (740990.250; Takara), and its concentration was measured using NanoDrop (2000/2000c Spectrophotometers, Thermo Fisher Scientific). The Illumina software package, “bcl2fastq”, was used for base-calling. The raw reads were mapped to the human reference genome sequence GRCh38 using TopHat (ver. 2.1.1) combined with Bowtie2 (ver. 2.3.4.1). Differential expression analysis was performed using the edgeR package, the enriched analysis was based on Gene Ontology (GO) database, and the pathway analysis by using Parametric Gene Set Enrichment Analysis (PGSEA) package (Ge et al., 2018, 2020).

### Statistical Analysis

All values are presented as the mean value of at least three independent experiments with standard deviation (SD). Two independent groups were compared using the Student’s t-test, while multiple group variance was compared using one-way analysis of variance (ANOVA), followed by Tukey’s test. *P*-values < 0.05 were considered significant for all statistical tests. Statistical analysis was performed using JMP Pro 14.0 (SAS).

### Data Availability

RNA-Seq data were deposited in the NCBI’s Gene Expression Omnibus (GEO series accession number GSE162707).

## Results

### Morphological Characteristics of hiPS-CMs Cultured on Different Topographic Patterns

**FIGURE 1:**
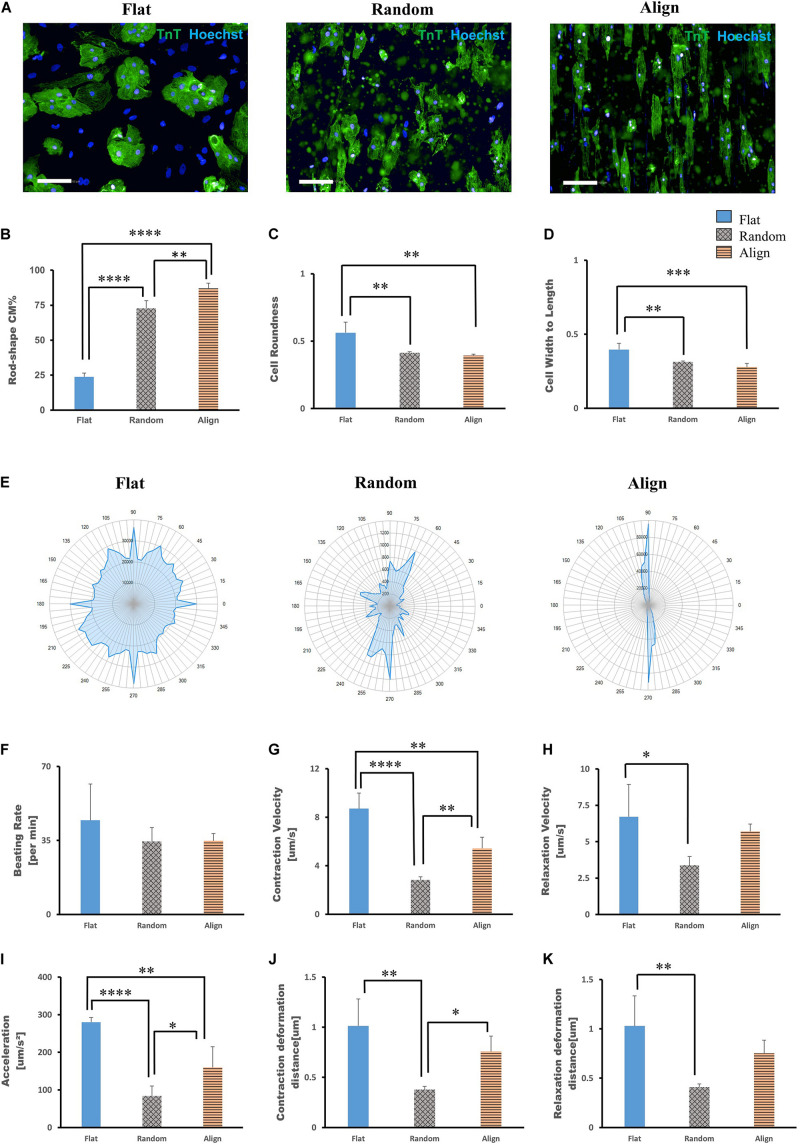
Morphological analysis of human induced pluripotent stem cell-derived cardiomyocytes (hiPS-CMs) on different patterns. **(A)** Immunofluorescence images of hiPS-CMs on flat, random, and aligned patterns at post-seeding day 7. hiPS-CMs were stained for cardiac troponin T (TnT, green) and the nuclei (Hoechst, blue). Scale bar, 100 μm. **(B)** Quantification of the proportion of rod-shaped hiPS-CMs on different patterns. The proportions of rod-shaped hiPS-CMs on random (72.8%) and aligned (87%) groups were both significantly higher than that in the flat group (23.7%). Error bars represent standard deviation (SD), ^∗∗^*P* < 0.01, ^****^P < 0.0001; *n* = 3. **(C)** The rod-shape was evaluated via cell roundness and cell width-to-length ratio analysis. Cell roundness close to 1 meant the cell shape was circular. hiPS-CMs on the flat pattern had a value of 0.56, whereas on the random and align patterns the values were both < 0.5 (0.41 and 0.39, respectively). Error bars represent standard deviation (SD), ^∗∗^*P* < 0.01, ^∗∗∗^*P* < 0.001; *n* = 3. **(D)** Cell width-to-length ratio close to 1 suggested round shape. The cell width-to-length ratio were 0.40, 0.31, and 0.28 for the flat, random, and align patterns, respectively. Error bars represent standard deviation (SD), ^∗∗^*P* < 0.01, ^∗∗∗^*P* < 0.001; *n* = 3. **(E–K)** Motion analysis of spontaneously beating hiPS-CMs. **(E)** Representative motion vector charts of hiPS-CMs on the flat, random, and aligned patterns. The scale on the dial represents each angle of contract direction, and the blue cover area reflects the combination of the vector orientation and the ratio of vectors within the same direction. hiPS-CMs on flat pattern showed multi-directional contraction; in contrast, hiPS-CMs on aligned pattern showed bi-directional contraction along the fiber orientation. hiPS-CMs on the random pattern also showed multi-directional contraction, but they were not as widely diffused as on the flat pattern. Comparison of contractile parameters. **(F)** Beating rate, **(G)** contraction velocity, **(H)** relaxation velocity, **(I)** contractile acceleration, **(J)** contraction deformation distance, and **(K)** relaxation deformation distance of hiPS-CMs on the flat, random, and aligned patterns. Data are presented as means ± standard deviation (SD). ^∗^*P* < 0.05, ^∗∗^*P* < 0.01, ^∗∗∗^*P* < 0.001, ^****^P < 0.0001; *n* = 3.

On post-differentiation day 28 − 30, highly purified hiPS-CMs were re-plated on three different topographic patterns: flat bottom, randomly oriented fiber matrix, and aligned fiber matrix (Supplementary Figure 1). After culturing for 1 week, the morphology of hiPS-CMs cultivated on different patterns showed significant differences. As shown in Figure 1A, the hiPS-CMs on the aligned fiber matrix were elongated and rod-shaped and were distributed along the fiber direction, whereas hiPS-CMs cultured on the other two patterns showed irregular shape and random distribution. In contrast to the hiPS-CMs on the flat bottom pattern that showed round shape and tended to aggregate, hiPS-CMs in the random group displayed multi-angular shape with smaller cell area. The cell shapes were quantified by analyzing immunofluorescence images using a high-content imaging system. Rod shape was evaluated via cell roundness and cell width-to-length ratio (Figures 1C,D). The cells with the morphology that met the following criteria, cell roundness ≤ 0.45 and cell width-to-length ratio of ≤ 0.5, was defined rod-shaped cell. Consequently, almost 90% and 70% hiPS-CMs were rod-shaped on the aligned and random fiber matrices, respectively; however, < 25% hiPS-CMs were rod-shaped on the flat bottom pattern (Figure 1B; flat(23.74 ± 2.71%) vs. random(72.76 ± 5.44%), P < 0.0001; flat vs. align(86.99 ± 3.77%), P < 0.0001; random vs. align, P < 0.01). To confirm our findings, we replicated the experiments using the human iPS cell line 253G1 derived cardiomyocytes. The immunofluorescence analysis showed similar results for rod-shape evaluation among the three patterns (Supplementary Figures 2A,B).

### Contractile Properties of hiPS-CMs

The contractile properties of hiPS-CMs were evaluated using the cell motion imaging system. In agreement with the morphology and distribution of hiPS-CMs in immunostaining images, hiPS-CMs in the aligned group showed bidirectional contraction; in contrast, hiPS-CMs in the flat and random groups performed multidirectional contraction (Figure 1E). hiPS-CMs cultured on these three patterns showed similar beating rates (Figure 1F; flat(44.65 ± 17.00/min) vs. random(34.55 ± 6.54/min) vs. align(34.8 ± 3.52/min), N.S. for among the three groups). hiPS-CMs on the flat bottom pattern showed faster contraction and relaxation velocities than those on the fiber-matrix patterns. hiPS-CMs in the random group showed the lowest contractile velocity and the smallest deformation during contraction (Figures 1F–K). The contractile analysis was also replicated with the 253G1 cell line of hiPS-CMs, and similar results were obtained (Supplementary Figures 2C–H).

### Electrophysiological Properties of hiPS-CMs

**FIGURE 2:**
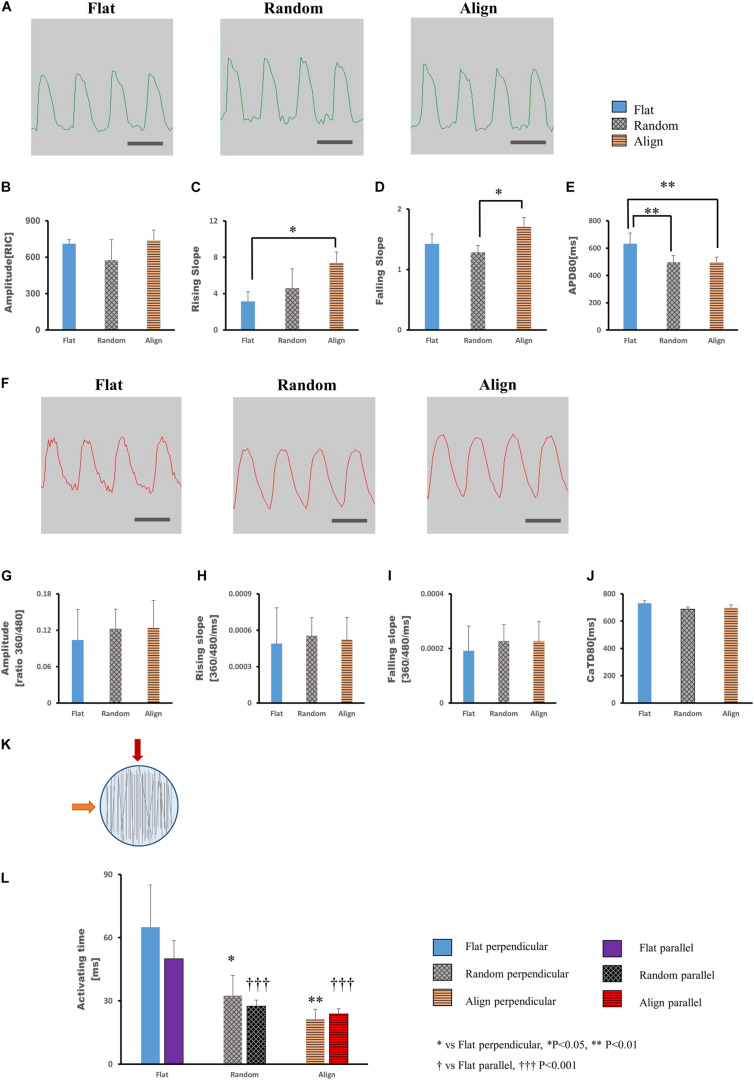
**(A–E)** Action potential of human induced pluripotent stem cell-derived cardiomyocytes (hiPS-CMs). **(A)** Representative waveforms for action potential of hiPS-CMs on flat, random, and aligned patterns. Time scale bar: 1 s. Comparison of the action potential parameters: **(B)** amplitude, **(C)** rising slope, **(D)** falling slope, and **(E)** action potential duration at 80% repolarization (APD80) of hiPS-CMs in the flat, random, and aligned groups. Data are presented as means ± standard deviation (SD). RIC, Relative Intensity Counts. ^∗^*P* < 0.05, ^∗∗^*P* < 0.01; *n* = 3. **(F–J)** Calcium transient of hiPS-CMs. **(F)** Representative waveforms for calcium transient of hiPS-CMs on flat, random, and aligned patterns. Time scale bar: 1 s. Comparison of calcium transient parameters: **(G)** amplitude, **(H)** rising slope, **(I)** falling slope, and **(J)** calcium transient duration at 80% relaxation (CaTD80) of hiPS-CMs in the flat, random, and aligned groups. The amplitude is measured by the ratio of fluorescence intensity at 360 nm and 480 nm. Data are presented as means ± standard deviation (SD). *n* = 3. The calcium transient parameters among the hiPS-CMs on different patterns did not vary significantly. **(K–L)** Evaluation of cardiac conductivity. **(K)** The schematic diagram for pacing direction. Solid red arrow indicates pacing parallel to the fiber orientation in the aligned pattern. The solid orange arrow indicates pacing perpendicular to the aligned fiber orientation. Parallel and perpendicular are relative to the fiber orientation in the aligned pattern, and accordingly, pacing at the same position in the flat and random groups. The light blue circle represents the well area; black lines represent nanofibers. **(L)** The conduction activating time of hiPS-CMs on different patterns. Irrespective of pacing parallel or perpendicular to the aligned fiber orientation, the activating time of hiPS-CMs in the random and aligned groups were both shorter than that in the flat group. In addition, on the same pattern, activating time did not vary when pacing along these two mutually vertical directions. Data are presented as means ± standard deviation (SD). ^∗^*P* < 0.05, ^∗∗^*P* < 0.01 for flat vs. random vs. aligned when pacing perpendicular to the aligned fiber orientation. ^†⁣†⁣†^*P* < 0.001 for flat vs. random vs. aligned group when pacing parallel to the aligned fiber orientation. *n* = 3.

After culturing on different patterns for 1 week, the hiPS-CMs showed significant differences in contractile properties. As contraction is involved with calcium fluctuation and action potential (AP), we also analyzed calcium transient and membrane potential using the FDSS/uCELL system. The AP amplitude did not vary significantly among the three patterns (Figures 2A,B), although the rising and falling slopes associated with the membrane potential were highest for the hiPS-CMs on the aligned fiber matrix (Figures 2C,D). The AP duration at 80% repolarization (APD80) was shortened in hiPS-CMs on random and aligned fiber matrices (Figure 2E; flat(633.08 ± 77.44 ms) vs. random(496.20 ± 50.43 ms), *P* < 0.01; flat vs. align(497.39 ± 37.68 ms), *P* < 0.01; random vs. align, P = N.S.). However, the parameters of calcium transient did not vary significantly among the hiPS-CMs cultured on these three patterns (Figures 2F–J), and 253G1-derived cardiomyocytes also showed similar results (Supplementary Figures 3A–D).

To investigate whether specific cell arrangement affected the conductivity, we assessed optical membrane potential imaging using the MiCAM02 imaging system. Considering the specific cell distribution on the aligned pattern, we performed pacing parallel (longitudinal) or perpendicular (transverse) to the orientation of the aligned fibers (Figure 2K) and at the same positions in the random and flat groups. As shown in Figure 2L (longitudinal: flat(50 ± 8.66 ms) vs. random(27.5 ± 2.89 ms), *P* < 0.001; flat vs. align(23.75 ± 2.5 ms), *P* < 0.001; random vs. align, P = N.S.; transverse: flat(65 ± 20 ms) vs. random(32.5 ± 9.57 ms), *P* < 0.05; flat vs. align(21.25 ± 4.79 ms), *P* < 0.01; random vs. align, P = N.S.), the activating time of hiPS-CMs on the fiber-matrix patterns was significantly shorter than that of hiPS-CMs on the flat bottom matrix, both along with the longitudinal and transverse directions. The longitudinal and transverse activating time in the same pattern did not vary significantly.

### Cardiac Gene Expression

**FIGURE 3:**
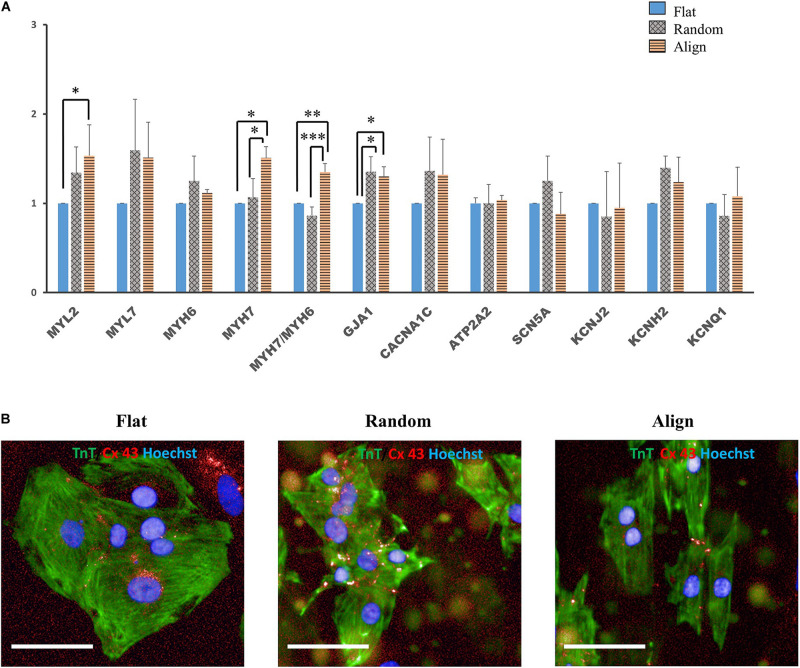
**(A)** Cardiac gene expression. Comparison of the expression of cardiac structural genes such as myosin light chain 2 (*MYL2*), myosin light chain 7 (*MYL7*), α-myosin heavy chain (*MYH6*), β-myosin heavy chain (*MYH7*), and gap junction connexin-43 (*GJA1*), calcium handling-associated genes such as L-type calcium channel (*CACNA1C*) and Sarco/endoplasmic reticulum Ca2 + -ATPase (*SERCA2*) (*ATP2A2*), conduction-related genes such as fast sodium (Na^+^) ion channel (*SCN5A*) and potassium (K^+^) ion channels, including K^+^ inward rectifier (*KCNJ2*), ether-a-go-go-related protein 1 (*KCNH2*), and K^+^ slow delayed rectifier channel (*KCNQ1*) of human induced pluripotent stem cell-derived cardiomyocytes (hiPS-CMs) on the flat, random, and aligned patterns. The expression levels of each gene were analyzed using quantitative reverse transcription-polymerase chain reaction (qRT-PCR) and normalized to 18S rDNA expression. ^∗^*P* < 0.05, ^∗∗^*P* < 0.01, ^∗∗∗^*P* < 0.001; *n* = 3. **(B)** Distribution of gap junction connexin-43 in hiPS-CMs. Immunofluorescence images of gap junction localization in hiPS-CMs on the flat, random, and aligned patterns. Images were obtained using a high content imaging system. hiPS-CMs were immunostained with TnT (green). The light red punctate stain represents connexin-43 (Cx-43). Cx-43 showed intercellular distribution in hiPS-CMs of the random and aligned groups, whereas, Cx-43 expression was negligible and was found at the intercellular spaces or at the cell borders in the flat group. Scale bar, 50 μm.

Despite the lack of a standard gene expression profile for defining the mature-like hiPS-CMs, we assessed the expression of several vital cardiac genes to determine changes at the molecular level in hiPS-CMs cultured on different substrates. α-myosin heavy chain (*MYH6*) and β-myosin heavy chain (*MYH7*) are well-known mature relevant cardiac genes. As shown in Figure 3A, *MYH7* expression and the ratio of *MYH7* to *MYH6* expression was highest in the aligned group. Expression of *MYL2*, a ventricular cardiac gene, in the aligned group was significantly higher than that in the flat group. The expression of genes involved in calcium handling, such as sarco/endoplasmic reticulum Ca2 + -ATPase (*SERCA2A*) (*ATP2A2*), and that of L-type calcium channel (*CACNA1C*) did not show significant difference among these three groups. The expression of genes related to cardiac AP, such as fast Na^+^ ion channel (*SCN5A*), K^+^ inward rectifier (*KCNJ2*), ether-a-go-go-related protein 1 (*KCNH2*), and K^+^ slow delayed rectifier channel (*KCNQ1*) did not differ among these three groups. The gap junction protein GJA1 was highly expressed in the fiber matrix pattern groups. Connexin-43 (Cx-43) mostly accumulated in the intercalated disks in the random or aligned group (Figure 3B), whereas its distribution in the flat group was not clear.

### Morphological and Functional Changes of hiPS-CMs After Reseeding on the Same Patterns

**FIGURE 4:**
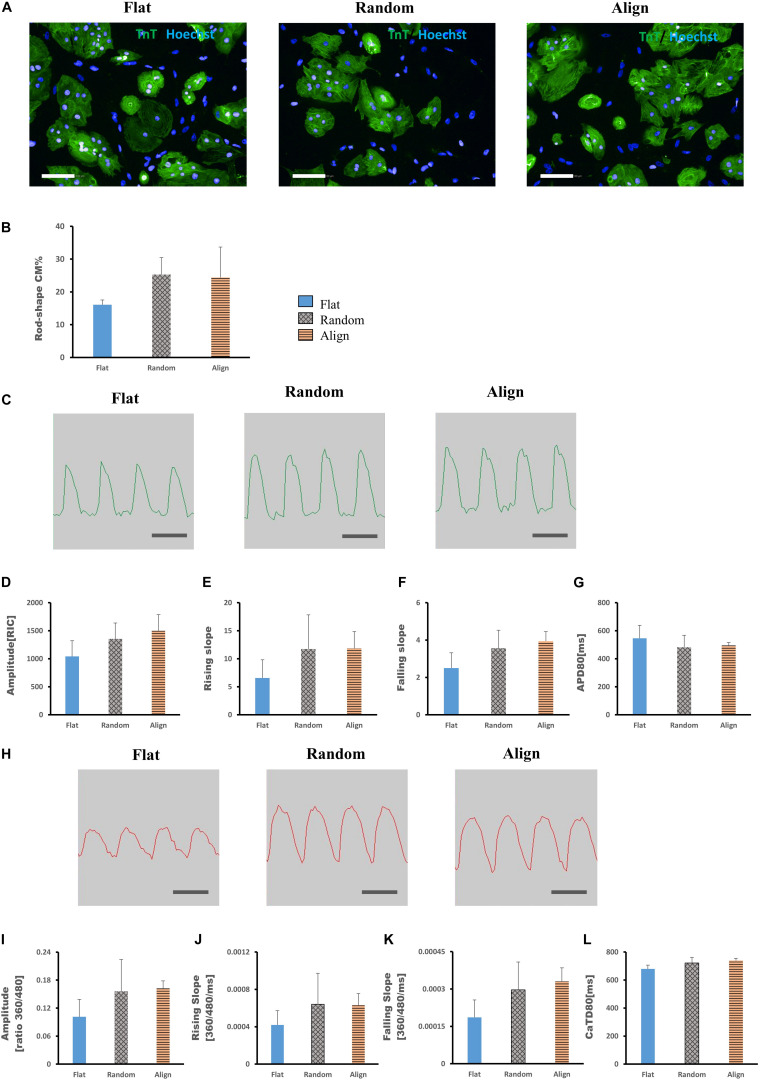
**(A,B)** Morphological changes of human induced pluripotent stem cell-derived cardiomyocytes (hiPS-CMs) after replating on non-scaffold (flat bottom) patterns. **(A)** hiPS-CMs pre-cultured on different patterns were individually replated on the flat pattern and cultured for one more week. Immunofluorescence images of hiPS-CMs pre-cultured on the flat, random, and aligned patterns. hiPS-CMs were stained for cardiac troponin T (TnT, green) and the nuclei (Hoechst, blue); scale bar, 100 μm. **(B)** Quantification of the proportion of rod-shaped hiPS-CMs. The proportion of rod-shaped hiPS-CMs did not vary significantly among these three groups. Error bars represent standard deviation (SD); data are presented as means ± SD; *n* = 3. **(C–G)** Action potential of hiPS-CMs after replating on non-scaffold (flat bottom) patterns. **(C)** Representative waveforms for action potential of hiPS-CMs pre-cultured on the flat, random, and aligned patterns. Time scale bar: 1 s. Action potential parameters: **(D)** amplitude, **(E)** rising slope, **(F)** falling slope, and **(G)** action potential duration at 80% repolarization (APD80) of hiPS-CMs did not vary significantly among these three groups. RIC, Relative Intensity Counts. Data are presented as means ± SD. *n* = 3. **(H–L)** Calcium transient of hiPS-CMs after replating on non-scaffold (flat bottom) patterns. **(H)** Representative waveforms for calcium transient of hiPS-CMs pre-cultured on the flat, random, and aligned patterns. Time scale bar: 1 s. Calcium transient parameters: **(I)** amplitude, **(J)** rising slope, **(K)** falling slope, and **(L)** calcium transient duration at 80% relaxation (CaTD80) of hiPS-CMs did not vary significantly among these three groups. The amplitude is measured by the ratio of fluorescence intensity at 360 nm and 480 nm. Data are presented as means ± standard deviation (SD). *n* = 3.

For determining whether the above morphological and functional changes in hiPS-CMs were induced by the culture substrates and disappeared after withdrawing the fiber substrates or were permanently encoded in the hiPS-CMs as inherent properties, hiPS-CMs were harvested from each pattern, replated onto the flat matrix, and incubated for another week. As shown in Figure 4A, after culturing on the flat bottom plates for one more week, the ratio of rod-shaped hiPS-CMs derived from fiber matrix patterns was still higher than that in the flat group, although the difference was not significant (Figure 4B; flat (16.09 ± 1.44%) vs. random(25.27 ± 5.23%) vs. align(24.39 ± 9.29%), P = N.S. for among the three groups).

**FIGURE 5:**
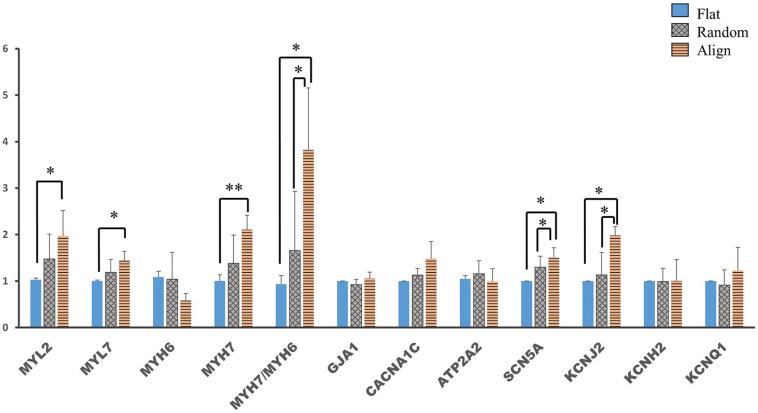
Cardiac gene expression of human induced pluripotent stem cell-derived cardiomyocytes (hiPS-CMs) after replating on non-scaffold (flat bottom) patterns. Cardiac gene expression was analyzed after the hiPS-CMs were replated on non-scaffold patterns and cultured for one more week. Comparison of the expression of cardiac structural genes such as myosin light chain 2 (*MYL2*), myosin light chain 7 (*MYL7*), α-myosin heavy chain (*MYH6*), β-myosin heavy chain (*MYH7*), and gap junction connexin-43 (*GJA1*), calcium handling-related genes such as L-type calcium channel (*CACNA1C*) and sarco/endoplasmic reticulum Ca2 + -ATPase (*SERCA2*) (*ATP2A2*), conduction-related genes such as fast sodium (Na^+^) ion channel (*SCN5A*) and potassium (K^+^) ion channels, including K^+^ inward rectifier (*KCNJ2*), ether-a-go-go-related protein 1 (*KCNH2*), and K^+^ slow delayed rectifier channel (*KCNQ1*) in hiPS-CMs pre-cultured on flat, random, and aligned patterns. Each gene expression level was normalized to *18S* rDNA expression. ^∗^*P* < 0.05, ^∗∗^*P* < 0.01; *n* = 3.

Membrane potential and calcium transient were also similar in terms of the wave amplitude, rising/falling slopes, and wave duration (Figures 4C–L). Interestingly, the assessment of cardiac gene expression showed that hiPS-CMs cultivated on different patterns partially retained the genetic differences (Figure 5). hiPS-CMs dissociated from aligned fiber matrix showed the highest expression of structural cardiac genes, *MYH7* and *MYL2*, and high expression ratio of *MYH7* to *MYH6*. In addition, the expression of *SCN5A* and *KCNJ2* increased in hiPS-CMs pre-cultured on the aligned pattern, whereas the expression levels of *ATP2A2, CACNA1C, GJA1, KCNH2*, and *KCNQ1* did not differ among the hiPS-CMs cultivated on different patterns. Furthermore, the random and align patterns-treated samples showed a higher ratio of *MYH7*/*MYH6* than the flat group at 2 weeks post separation (Supplementary Figure 5, flat(1 ± 0) vs. random(3.026 ± 0.11), *P* < 0.01; flat(1 ± 0) vs. align(3.410 ± 0.92), *P* < 0.01; random vs. align, P = N.S.). We also conducted the cardiac gene expression analysis using 253G1 hiPS-CMs. Similarly, hiPS-CMs cultured on the aligned pattern for 1 week showed significantly higher levels of *MLC2*, *MYH7*, and *GJA1* than the flat and random groups did. Furthermore, *MYL7*, *ATP2A2*, and *SCN5A* also showed significantly higher expression compared to flat and random groups (Supplementary Figure 4A). After replating on non-scaffold (flat bottom) patterns for 1 week, consistent with 201B7 results, there is a significantly higher expression of MYL2 and MYH7 in the align group than in the flat and random groups (Supplementary Figure 4B).

### Differential Gene Expression and Enriched Pathway Analysis

**FIGURE 6:**
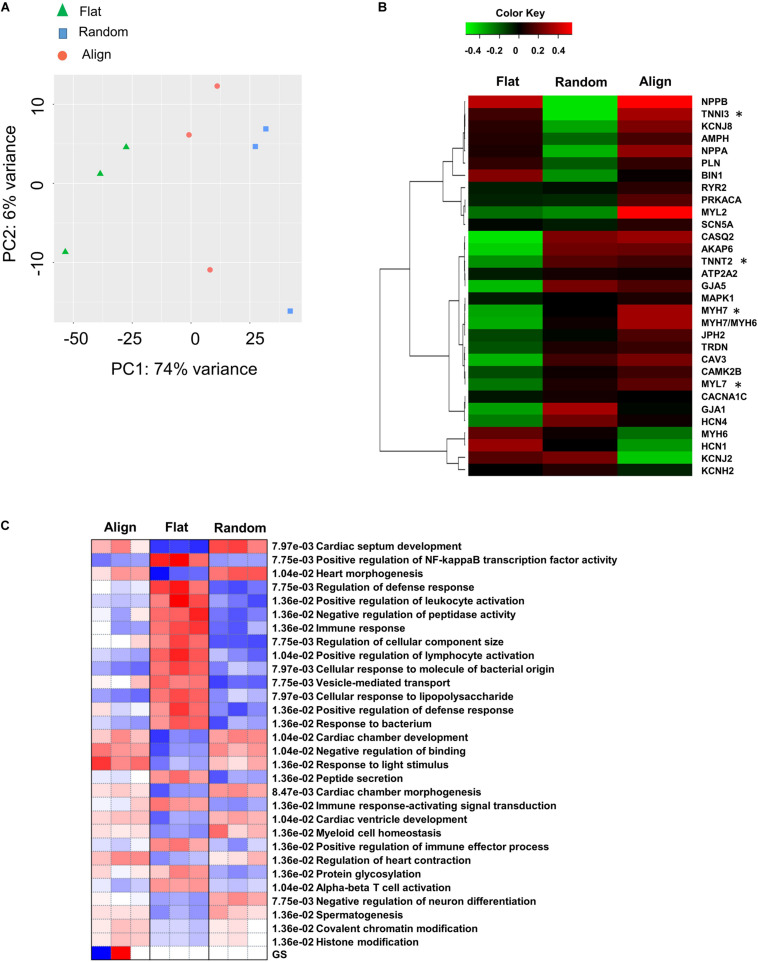
Differential gene expression. **(A)** Principal component analysis (PCA) for flat, random, and align groups. Green triangles, blue squares, and pink circles represent the flat, random, and align groups, respectively. False discovery rate (FDR) < 0.05 (*n* = 3). **(B)** Heatmap for cardiac mature relevant genes on flat, random, and align groups. The data are represented by mean values for three biological replicate samples for each group. *FDR < 0.05. Cut-off Z score: -2 < z < 2 (*n* = 3). **(C)** Pathways analysis on flat, random, and align groups using the PGSEA algorithm. Pathway significance cut-off FDR < 0.05 (*n* = 3).

To get a comprehensive understanding of the genome-wide expression dynamics under topographic stimulation and, more importantly, to obtain insights into the underlying mechanisms of the developmental effects driven by topographic stimuli, we performed RNA-seq of hiPS-CMs after culturing on flat, random, or align patterns for 1 week. Principal component analysis (PCA) revealed that the most variance between the RNA-seq samples from flat and random patterns (Figure 6A). Moreover, the flat group samples occupied most of the enrichment gene expression, as shown by the Differentially expressed genes(DEGs) detected with the ‘DESeq2’ package and the hierarchical clustering (Supplementary Figure 6A). Analysis of the enriched DEGs based on GO biological processes revealed that the upregulated genes in the flat group were mainly related to the regulation of cell movement, including extracellular matrix organization, cell adhesion, and cell migration. Well-known cardiac maturation markers (Ronaldson-Bouchard et al., 2018), such as MYH7 and TNNI3, were found to be significantly upregulated in the align group, which was consistent with the qPCR analysis results. Moreover, cardiac structural (*MLC2*, *TNNT2*, *GJA1*) and calcium handling relevant genes (*CASQ2*, *CAMK2B*, *CAV3*) also showed a higher expression in align group than that in the flat group (Figure 6B). The most upregulated gene sets in the align group included those related to cardiac development and heart morphogenesis pathways, negative regulation of binding, and microtubule-based processes (Figure 6C). In contrast, Members of the keratin(KRT) gene family, which play a role in cell movement, were found to be involved in the relatively downregulated-gene enriched pathways.

## Discussion

In this study, we generated a well-organized and aligned hiPS-derived cardiac construct by culturing hiPS-CMs on anisotropic fiber matrix, which geometrically mimics the native heart tissue in only few days. Several strategies were used for constructing anisotropic cardiac tissues, ranging from the design of structures (concave-convex mode and oriented fiber like matrix) to the use of different types of substrate materials (polydimethylsiloxane, silk fibroin, and electrospun fibrous) (Stoppel et al., 2015; Han et al., 2016; Pilarczyk et al., 2016). Compared to the anisotropic microgroove modes, the nanofiber patterns used in this study maintained the intercellular space and increased intercellular connection, which is similar to the microenvironment of native heart tissue. Furthermore, hiPS-CMs grown on the fiber substrates tightly integrate with the nanofibers over time, which may decrease contraction movement. However, it is noteworthy that the motion analysis of contraction kinetic parameters was the mean value of measurements from every direction and not along one of the specific orientations. Considering this, the contraction velocity along the fiber orientation may be faster in the align group than that in the flat group. Regarding energy consumption, hiPS-CMs on the aligned pattern showed bidirectional contractions, which may reduce energy loss. In contrast, the hiPS-CMs in the flat and random groups showed multidirectional contractions. Furthermore, upregulation of the mature isoforms of the sarcomeric gene *MYH7* in the aligned hiPS-CMs suggests that the bidirectional contractions of hiPS-CMs may be a mechanical stimulation that contributes to hiPS-CM maturation.

Cardiac contraction is accompanied by calcium cycling in each cardiomyocyte, and the immaturity of hiPS-CMs is manifested in low calcium release and reuptake kinetics (Denning et al., 2016). However, the hiPS-CMs on the three patterns did not differ significantly in calcium transient or the expression of calcium handling-associated genes such as *ATP2A2* and *CACNA1C*, indicating that relative to the morphological changes, the development of calcium transient in hiPS-CMs required time and was negligibly affected by matrix geography. AP is one of the most important electrophysiological properties of multiple ion currents. hiPS-CMs showed lower upstroke velocity of AP and longer action potential duration (APD) than those of adult ventricular cardiomyocytes due to the low density of functional mature ion channels (Denning et al., 2016; Kolanowski et al., 2017; Scuderi and Butcher, 2017). In our study, the upslope of AP in hiPS-CMs of the aligned group was more striking than that of the flat group; furthermore, the APD80 of the hiPS-CMs of the aligned group or random group was significantly shorter than that of the flat group. Interestingly, the expression of the cardiac AP-related genes, namely, *SCN5A*, *KCNJ2*, *KCNH2*, and *KCNQ1*, did not show the corresponding difference. Based on these results, we speculated that at least for cardiac AP, the aligned cell distribution close relative to the change of electrophysiological properties of hiPS-CMs.

Cardiac conductivity, another essential property of CMs, is also an important parameter for estimating hiPS-CM maturation. The fatal cardiac arrhythmia caused by cardiac infarction or any other induced myocardial damages is usually attributed to cardiac conduction abnormalities, which is partly due to the disorganization of cardiac cells in the injured area. With the exception of the low density of ion channels, deficiency of conductivity in immature hiPS-CMs is due to the negligible expression of gap junctions and the distribution of gap junctions at the cell circumference rather than at the intercalated disks (Denning et al., 2016). In the present study, the cardiac activating time of hiPS-CMs in the scaffold patterns was significantly shorter than that in the flat pattern, suggesting that the conductivity of hiPS-CMs on the aligned or random pattern was faster than that on the flat pattern. Immunostaining images showed that most Cx-43 was located at the intercalated disks of hiPS-CMs on the random and aligned patterns, whereas, the negligible amount of Cx-43 was observed on the flat pattern. In agreement with the result of Cx-43 immunostaining, the expression of the gap junction Cx-43-encoding gene, *GJA1*, in both the randomized and aligned groups was higher than that in the flat group. The above results indicate that the acceleration of cardiac conductivity may benefit from the accumulation of more gap junctions at the intercellular locations. Interestingly, the conduction velocities parallel and perpendicular to the fiber orientation in the aligned substrate did not differ. As the CMs in the ventricular wall are not uniformly aligned, it is possible that overlapping CMs laminate with the aligned myocytes layer by layer (Streeter et al., 1969; LeGrice et al., 1995). It is noteworthy that CM orientation affects both electrical activation and excitation propagation (Hooks et al., 2002). In addition to CMs, aligned cell arrangement also affects the induction of cardiac differentiation (Pijnappels et al., 2008). Here, the similar conduction velocities parallel and perpendicular to the CM orientation may be attributed to the immature hiPS-CMs or non-CMs and also to the heterogeneous distribution of the gap junctions.

Although a previous study suggested that the cell shape has profound effects on the function and molecular expression in cardiomyocytes (Haftbaradaran Esfahani et al., 2019), the extent to which the benefits—driven by morphological changes—on hiPS-CMs last remained unclear. Our study first investigated the memory of iPS-CMs for the aligned pattern-induced facilitation of maturity. The cells were removed from the different substrates and were then replated on the flat pattern for an additional week. Unfortunately, hiPS-CMs pre-cultured on the aligned matrix were round-shaped and randomly oriented, similar to the other two groups. Furthermore, AP and calcium transient did not differ among these three groups. However, the restoration of the morphological and functional changes indicates that cell orientation significantly affects the electrical properties of hiPS-CMs. Surprisingly, cardiac gene expression showed that hiPS-CMs pre-cultured on the aligned pattern were considerably more mature than those derived from the flat group and that the difference was even more significant than that in the pre-culture stage. Thus, the round shape and the change in AP were not because of the degeneration of hiPS-CMs; conversely, hiPS-CM maturation was still underway. In addition, the hiPS-CMs pre-cultured on the aligned pattern somehow retained the memory of the beneficial effects from topographic induced maturation, and maintained during development even without the fiber matrix.

To obtain insights into the underlying molecular pathways/signaling involved in the facilitation of hiPS-CM maturation driven by specific topographic stimuli, we performed RNA-seq on hiPS-CM samples after different patterns culturing. The RNA-seq samples of the flat group occupied most of the enrichment gene expression landscape (Supplementary Figure 6A), with the related pathways being mostly involved in the regulation of cell movement. This finding suggested that, without the scaffold support, the cells could induce the self-synthesis of the extracellular matrix; thus, cells on the flat pattern may induce the facilitation of cell proliferation by controlling the extracellular matrix remodeling process (Cui et al., 2018). Moreover, the hierarchical clustering results revealed that several cardiac mature markers had a higher expression in the align group (Figure 6B). The switch of predominant contractile isoforms between MYH6/MYH7 and TNNI2/TNNI3 is a hallmark of cardiomyocyte maturation (Taegtmeyer et al., 2010; Ames et al., 2013). RNA-seq data revealed significantly elevated expression of *MYH7* and *TNNI3* in the align group samples. In addition to providing substantial cell developmental molecular information, the differential GO pathway enrichment among hiPS-CM samples from flat, random, and align patterns also provided detailed insights on the underlying mechanisms involved in topographic stimuli-derived maturation of hiPS-CMs. The most upregulated gene sets related pathways in the align group were closely related to *FOS* and *JUN* families, which are both known to be associated with the regulation of stimuli-induced cell responses (Singal et al., 2009). Similar enriched pathways have also been seen in the random group samples; thus, it could also be related to substrate stimulation. Upon overlapping the upregulated genes in the align pattern compared to the flat or random group, six genes were found to be specifically upregulated in align vs. flat (Supplementary Figure 6B, including *F13A1*, *XIRP2*, *TTC29*, *PTPRH*, *TULP1*, and *LINC00702*). In particular, Protein Tyrosine Phosphatase Receptor Type H (PTPRH) was identified, which is a member of the protein tyrosine phosphatase (PTP) family that is involved in the regulation of various cellular processes including cell differentiation and mitotic cycle (O’Leary et al., 2016). Naturally, during heart development, CMs experience a transition from cell proliferation to cellular hypertrophy. Based on our findings, the aligned topographic cell arrangement may be related to the regulation of CM maturation by modulating the cell cycle. Therefore, it is relevant to investigate the underlying signaling involved in the regulation of cell growth that is driven by the aligned cell organization.

## Conclusion

**FIGURE 7:**
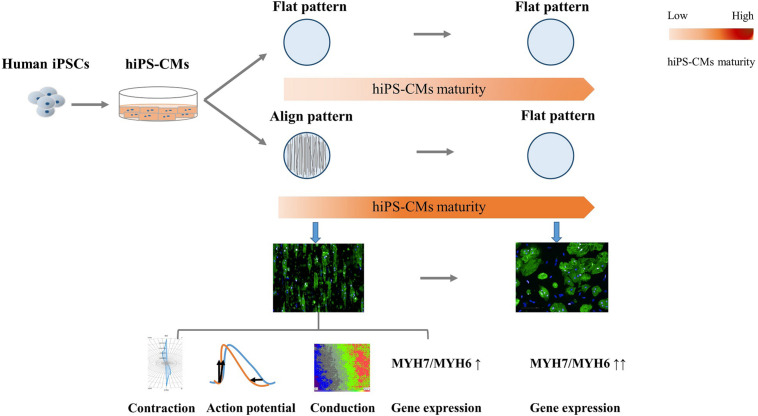
Schema for the present study. Human induced pluripotent stem cell-derived cardiomyocytes (hiPS-CMs) cultivated on aligned pattern showed more mature-like properties than that on non-scaffold (flat) pattern, including elongated rod shape, bidirectional contraction, efficient action potential, faster conduction velocity, and elevated cardiac maturity-related gene expression. The morphological and electrophysiological advantages diminished once the hiPS-CMs were freed from the aligned scaffold, although the molecular developmental properties were preserved.

In summary, in the present study, we constructed geometric native-like hiPS-cardiac tissue by culturing cardiomyocytes on well-oriented nanofibers. In addition to morphological improvement, which involved a change from the round irregular shape and random distribution to elongated rod shape and good organization, respectively, the CMs displayed functional improvements, including more efficient AP and faster conduction velocity (Figure 7). The “passive” rod-shaped hiPS-CMs were not equivalent to the adult ventricular cardiomyocytes, and these morphological changes diminished after withdrawing the exterior support. This may be analogous to a case where an “infant” wearing “adult clothes” looks mature; however, the “adult clothes,” i.e., rod shape and alignment, facilitate hiPS-CM maturation. In other words, in addition to the fact that mature CMs are more functional than their immature counterparts, the functional environment also promotes CM maturation. Importantly, these functional improvements persist during hiPS-CM development even without exterior support. This may also circumvent the concerns regarding the quality of delivered isolated cells, which may be used instead of transplanting an intact tissue, thereby providing more options for cell therapy.

## Data Availability Statement

RNA-Seq data were deposited in the NCBI’s Gene Expression Omnibus, and are accessible through GEO Series accession number GSE162707 (https://www.ncbi.nlm.nih.gov/geo/query/acc.cgi?acc=GSE162707).

## Ethics Statement

The studies involving human participants were reviewed and approved by the Institutional Review Board of Osaka University [Approval number 13254 (829-1)-3]. The patients/participants provided their written informed consent to participate in this study.

## Author Contributions

JL and J-KL conceptualized the study, designed and performed the experiments, processed the data, and wrote the manuscript. KeM, YK, HN, ST, HY, KiM, and YS participated in the analysis and reviewed the manuscript. YS supervised the study. All the authors qualified for authorship and approved the final version of the manuscript, contributed to the article, and approved the submitted version.

## Conflict of Interest

The authors declare that the research was conducted in the absence of any commercial or financial relationships that could be construed as a potential conflict of interest.

## Abbreviations

AP: action potential
APD80: AP duration at 80% repolarization
CACNA1C: L-type calcium channel
Cx-43: connexin-43
DEG: differentially expressed gene
GJA1: gap junction connexin-43
GO: gene ontology
hiPS-CMs: human induced pluripotent stem cell-derived cardiomyocytes
KCNH2: ether-a-go-go-related protein 1
KCNJ2: potassium inward rectifier
KCNQ1: potassium slow delayed rectifier channel
KRT: keratin
qRT-PCR: quantitative reverse transcription-polymerase chain reaction
MYH6: α -myosin heavy chain
MYH7: β -myosin heavy chain
MYL2: myosin light chain 2
MYL7: myosin light chain 7
PCA: principal component analysis
PGSEA: parametric gene set enrichment analysis
PTP: protein tyrosine phosphatase
PTPRH: protein tyrosine phosphatase receptor type H
SERCA2/ATP2A2: Sarco/endoplasmic reticulum calcium-ATPase
SCN5A: fast sodium ion channel.

## References

[B1] Ames E. G. Lawson M. J. Mackey A. J. Holmes J. W. (2013). Sequencing of mRNA identifies re-expression of fetal splice variants in cardiac hypertrophy. *J. Mol. Cell. Cardiol.* 62 99–107. 10.1016/j.yjmcc.2013.05.004 23688780 PMC3735658

[B2] Anderson D. Self T. Mellor I. R. Goh G. Hill S. J. Denning C. (2007). Transgenic enrichment of cardiomyocytes from human embryonic stem cells. *Mol. Ther.* 15 2027–2036. 10.1038/sj.mt.6300303 17895862

[B3] Chun Y. W. Voyles D. E. Rath R. Hofmeister L. H. Boire T. C. Wilcox H. (2015). Differential responses of induced pluripotent stem cell-derived cardiomyocytes to anisotropic strain depends on disease status. *J. Biomech.* 48 3890–3896. 10.1016/j.jbiomech.2015.09.028 26476764 PMC4655132

[B4] Cui M. Wang Z. Bassel-Duby R. Olson E. N. (2018). Genetic and epigenetic regulation of cardiomyocytes in development, regeneration and disease. *Development* 145:dev171983. 10.1242/dev.171983 30573475 PMC6307883

[B5] Denning C. Borgdorff V. Crutchley J. Firth K. S. A. George V. Kalra S. (2016). Cardiomyocytes from human pluripotent stem cells: from laboratory curiosity to industrial biomedical platform. *Biochim. Biophys. Acta* 1863 1728–1748. 10.1016/j.bbamcr.2015.10.014 26524115 PMC5221745

[B6] Engels M. C. Rajarajan K. Feistritzer R. Sharma A. Nielsen U. B. Schalij M. J. (2014). Insulin-like growth factor promotes cardiac lineage induction in vitro by selective expansion of early mesoderm. *Stem Cells* 32 1493–1502. 10.1002/stem.1660 24496962 PMC4037352

[B7] Ge S. X. Jung D. Yao R. (2020). ShinyGO: a graphical gene-set enrichment tool for animals and plants. *Bioinformatics* 36 2628–2629. 10.1093/bioinformatics/btz931 31882993 PMC7178415

[B8] Ge S. X. Son E. W. Yao R. (2018). iDEP: an integrated web application for differential expression and pathway analysis of RNA-Seq data. *BMC Bioinform.* 19:534. 10.1186/s12859-018-2486-6 30567491 PMC6299935

[B9] Gersh B. J. Sliwa K. Mayosi B. M. Yusuf S. (2010). Novel therapeutic conceptsThe epidemic of cardiovascular disease in the developing world: global implications. *Eur. Heart J.* 31 642–648. 10.1093/eurheartj/ehq030 20176800

[B10] Haftbaradaran Esfahani P. ElBeck Z. Sagasser S. Li X. Hossain M. B. Talukdar H. A. (2019). Cell shape determines gene expression: cardiomyocyte morphotypic transcriptomes. *Basic Res. Cardiol.* 115:7. 10.1007/s00395-019-0765-7 31872302 PMC6928094

[B11] Han J. Wu Q. Xia Y. Wagner M. B. Xu C. (2016). Cell alignment induced by anisotropic electrospun fibrous scaffolds alone has limited effect on cardiomyocyte maturation. *Stem Cell Res.* 16 740–750. 10.1016/j.scr.2016.04.014 27131761 PMC4903921

[B12] Hooks D. A. Tomlinson K. A. Marsden S. G. LeGrice I. J. Smaill B. H. Pullan A. J. (2002). Cardiac microstructure: implications for electrical propagation and defibrillation in the heart. *Circ. Res.* 91 331–338. 10.1161/01.res.0000031957.70034.89 12193466

[B13] Ichimura H. Shiba Y. (2017). Recent progress using pluripotent stem cells for cardiac regenerative therapy. *Circ. J.* 81 929–935. 10.1253/circj.CJ-17-0400 28603177

[B14] Iseoka H. Miyagawa S. Fukushima S. Saito A. Masuda S. Yajima S. (2018). Pivotal role of non-cardiomyocytes in electromechanical and therapeutic potential of induced pluripotent stem cell-derived engineered cardiac tissue. *Tissue Eng. Part A* 24 287–300. 10.1089/ten.TEA.2016.0535 28498040 PMC5792250

[B15] Kim D.-H. Kshitiz, Smith R. R. Kim P. Ahn E. H. Kim H.-N. (2012). Nanopatterned cardiac cell patches promote stem cell niche formation and myocardial regeneration. *Integr. Biol. (Camb.)* 4 1019–1033. 10.1039/c2ib20067h 22890784

[B16] Kolanowski T. J. Antos C. L. Guan K. (2017). Making human cardiomyocytes up to date: derivation, maturation state and perspectives. *Int. J. Cardiol.* 241 379–386. 10.1016/j.ijcard.2017.03.099 28377185

[B17] LeGrice I. J. Smaill B. H. Chai L. Z. Edgar S. G. Gavin J. B. Hunter P. J. (1995). Laminar structure of the heart: ventricular myocyte arrangement and connective tissue architecture in the dog. *Am. J. Physiol.* 269 H571–H582. 10.1152/ajpheart.1995.269.2.H571 7653621

[B18] Nakanishi H. Lee J.-K. Miwa K. Masuyama K. Yasutake H. Li J. (2019). Geometrical patterning and constituent cell heterogeneity facilitate electrical conduction disturbances in a human induced pluripotent stem cell-based platform: an in vitro disease model of atrial arrhythmias. *Front. Physiol.* 10:818. 10.3389/fphys.2019.00818 31316396 PMC6610482

[B19] O’Leary N. A. Wright M. W. Brister J. R. Ciufo S. Haddad D. McVeigh R. (2016). Reference sequence (RefSeq) database at NCBI: current status, taxonomic expansion, and functional annotation. *Nucleic Acids Res.* 44 D733–D745. 10.1093/nar/gkv1189 26553804 PMC4702849

[B20] Pijnappels D. A. Schalij M. J. Ramkisoensing A. A. van Tuyn J. de Vries A. A. F. van der Laarse A. (2008). Forced alignment of mesenchymal stem cells undergoing cardiomyogenic differentiation affects functional integration with cardiomyocyte cultures. *Circ. Res.* 103 167–176. 10.1161/CIRCRESAHA.108.176131 18556577

[B21] Pilarczyk G. Raulf A. Gunkel M. Fleischmann B. K. Lemor R. Hausmann M. (2016). Tissue-mimicking geometrical constraints stimulate tissue-like constitution and activity of mouse neonatal and human-induced pluripotent stem cell-derived cardiac myocytes. *J. Funct. Biomater* 7:1. 10.3390/jfb7010001 26751484 PMC4810060

[B22] Ronaldson-Bouchard K. Ma S. P. Yeager K. Chen T. Song L. Sirabella D. (2018). Advanced maturation of human cardiac tissue grown from pluripotent stem cells. *Nature* 556 239–243. 10.1038/s41586-018-0016-3 29618819 PMC5895513

[B23] Ruan J.-L. Tulloch N. L. Razumova M. V. Saiget M. Muskheli V. Pabon L. (2016). Mechanical stress conditioning and electrical stimulation promote contractility and force maturation of induced pluripotent stem cell-derived human cardiac tissue. *Circulation* 134 1557–1567. 10.1161/CIRCULATIONAHA.114.014998 27737958 PMC5123912

[B24] Sakai T. Naito A. T. Kuramoto Y. Ito M. Okada K. Higo T. (2018). Phenotypic screening using patient-derived induced pluripotent stem cells identified Pyr3 as a candidate compound for the treatment of infantile hypertrophic cardiomyopathy. *Int. Heart J.* 59 1096–1105. 10.1536/ihj.17-730 30101858

[B25] Scuderi G. J. Butcher J. (2017). Naturally engineered maturation of cardiomyocytes. *Front. Cell Dev. Biol.* 5:50. 10.3389/fcell.2017.00050 28529939 PMC5418234

[B26] Singal T. Dhalla N. S. Tappia P. S. (2009). Regulation of c-Fos and c-Jun gene expression by phospholipase C activity in adult cardiomyocytes. *Mol. Cell. Biochem.* 327 229–239. 10.1007/s11010-009-0061-1 19225867

[B27] Stoppel W. L. Hu D. Domian I. J. Kaplan D. L. Black L. D. III (2015). Anisotropic silk biomaterials containing cardiac extracellular matrix for cardiac tissue engineering. *Biomed. Mater.* 10:34105. 10.1088/1748-6041/10/3/034105 PMC4417360 25826196

[B28] Streeter D. D. J. Spotnitz H. M. Patel D. P. Ross J. J. Sonnenblick E. H. (1969). Fiber orientation in the canine left ventricle during diastole and systole. *Circ. Res.* 24 339–347. 10.1161/01.res.24.3.339 5766515

[B29] Sun N. Yazawa M. Liu J. Han L. Sanchez-Freire V. Abilez O. J. (2012). Patient-specific induced pluripotent stem cells as a model for familial dilated cardiomyopathy. *Sci. Transl. Med.* 4:130ra47. 10.1126/scitranslmed.3003552 22517884 PMC3657516

[B30] Taegtmeyer H. Sen S. Vela D. (2010). Return to the fetal gene program. *Ann. N. Y. Acad. Sci.* 1188 191–198. 10.1111/j.1749-6632.2009.05100.x 20201903 PMC3625436

[B31] Tohyama S. Hattori F. Sano M. Hishiki T. Nagahata Y. Matsuura T. (2013). Distinct metabolic flow enables large-scale purification of mouse and human pluripotent stem cell-derived cardiomyocytes. *Cell Stem Cell* 12 127–137. 10.1016/j.stem.2012.09.013 23168164

[B32] Veerman C. C. Kosmidis G. Mummery C. L. Casini S. Verkerk A. O. Bellin M. (2015). Immaturity of human stem-cell-derived cardiomyocytes in culture: fatal flaw or soluble problem? *Stem Cells Dev.* 24 1035–1052. 10.1089/scd.2014.0533 25583389

[B33] Yang X. Rodriguez M. Pabon L. Fischer K. A. Reinecke H. Regnier M. (2014). Tri-iodo-l-thyronine promotes the maturation of human cardiomyocytes-derived from induced pluripotent stem cells. *J. Mol. Cell. Cardiol.* 72 296–304. 10.1016/j.yjmcc.2014.04.005 24735830 PMC4041732

[B34] Yazawa M. Hsueh B. Jia X. Pasca A. M. Bernstein J. A. Hallmayer J. (2011). Using induced pluripotent stem cells to investigate cardiac phenotypes in Timothy syndrome. *Nature* 471 230–234. 10.1038/nature09855 21307850 PMC3077925

[B35] Yoshida Y. Yamanaka S. (2017). Induced pluripotent stem cells 10 years later: for cardiac applications. *Circ. Res.* 120 1958–1968. 10.1161/CIRCRESAHA.117.311080 28596174

[B36] Zhang J. (2015). Engineered tissue patch for cardiac cell therapy. *Curr. Treat. Options Cardiovasc. Med.* 17:399. 10.1007/s11936-015-0399-5 26122908 PMC4676725

