# Supplementary material for: Scaffold-Mediated Developmental Effects on Human Induced Pluripotent Stem Cell-Derived Cardiomyocytes Are Preserved After External Support Removal
Source: Front Cell Dev Biol. 2021 Feb 15;9:591754. doi: 10.3389/fcell.2021.591754 (PMC7917244; doi:10.3389/fcell.2021.591754)
Supplement: Supplementary file 1 [file Data_Sheet_1.pdf]

Supplementary Figure 1

A

B

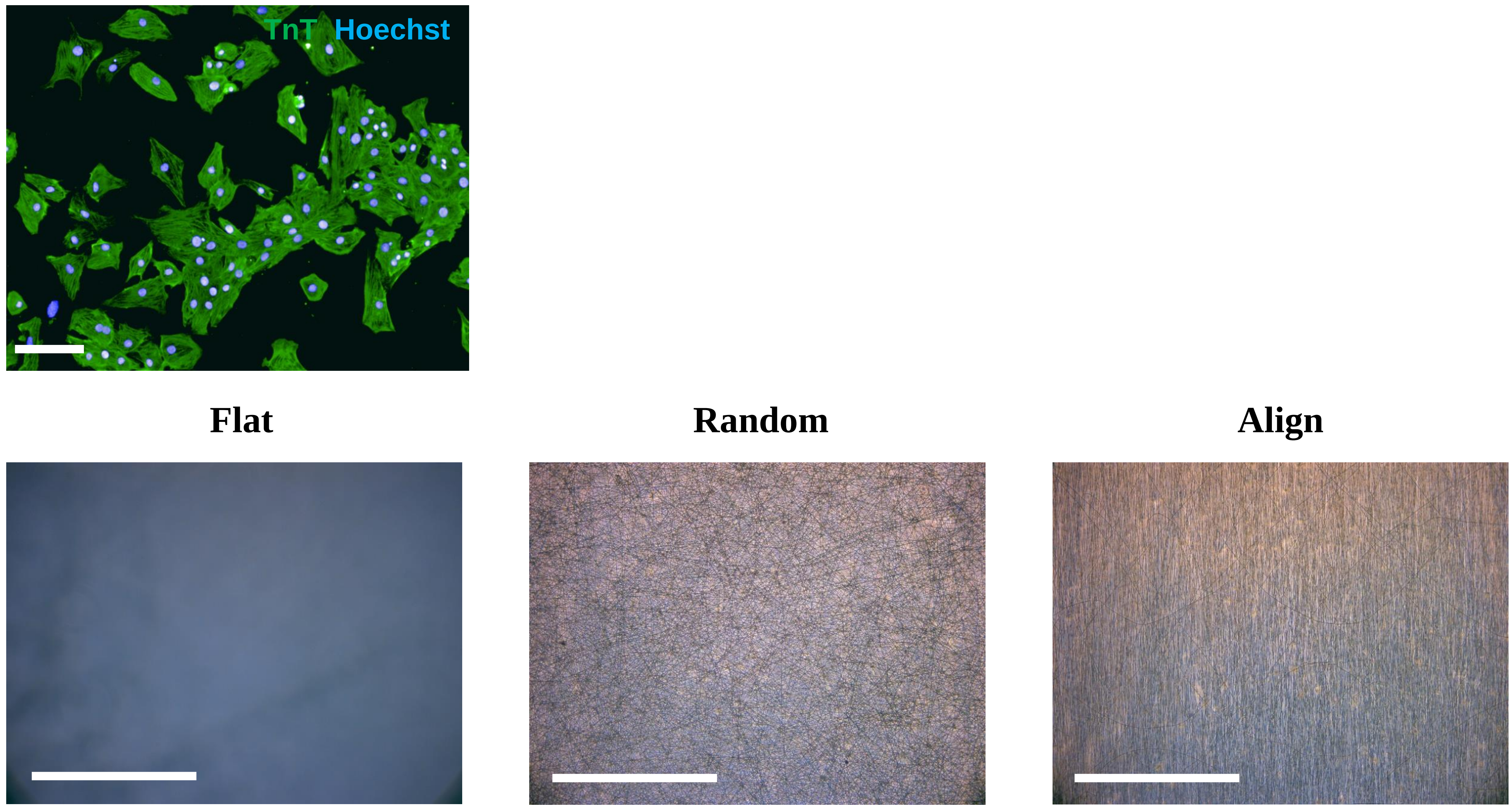

Supplementary Figure 1. (A) Immunofluorescence image of purified human induced pluripotent stem cell-derived cardiomyocytes (hiPS-CMs). hiPS-CMs were stained with cardiac troponin-T (TnT, green) and nuclei (Hoechst, blue). Scale bar, 100 µm. The proportion of TnT positive cardiomyocytes was 94.75 ± 1.38%. Data are presented as means ± standard deviation (SD), n = 3. **(B)** Light microscope images of the flat bottom (flat), random oriented fiber substrate (random), anisotropic aligned fiber substrate (align). Scale bar, 1 mm.

Supplementary Figure 2

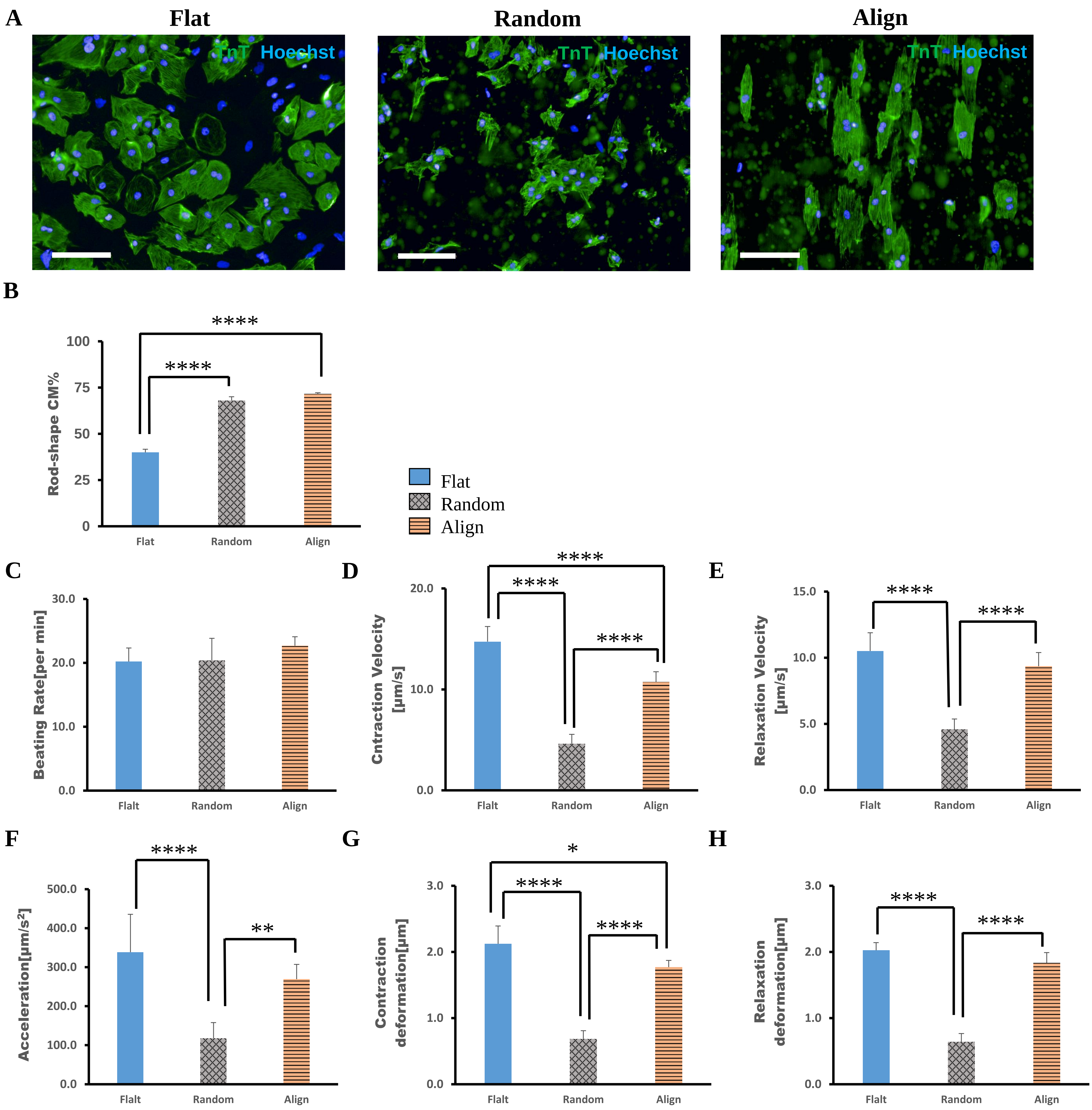

Supplementary Figure 2. (A, B) Morphological analysis of 253G1 human induced pluripotent stem cell-derived cardiomyocytes (hiPS-CMs) on different patterns. (A) Immunofluorescence images of hiPS-CMs on flat, random, and align patterns 7 days post-seeding. hiPS-CMs were stained for cardiac troponin T (TnT, green) and the nuclei (Hoechst, blue). Scale bar, 100 μm. **(B)** Quantification of the proportion of rod-shaped hiPS-CMs on the different patterns. The proportion of rod-shaped hiPS-CMs in random and align groups was significantly higher than the proportion in the flat group (flat, 40.0±1.67% vs random, 67.9±2.11%; align, 71.5±0.71%). Error bars represent standard deviation (SD), ****P < 0.0001; n = 3. **(C-H)** Motion analysis of spontaneously beating 253G1 hiPS-CMs. Comparison of contractile parameters of hiPS-CMs on the flat, random, and align patterns; **(C)** beating rate – flat, 20.2±2.1/min; random, 20.4±3.5/min; align, 22.7±1.4/min; **(D)** contraction velocity – flat, 14.7±1.5 μm/s; random, 4.6±0.9 μm/s; align, 10.7±1.0 μm/s; **(E)** relaxation velocity – flat, 10.5±1.4 μm/s; random, 4.6±0.8 μm/s; align, 9.4±1.0 μm/s; **(F)** contractile acceleration – flat, 338.7±96.9 μm/s^2; random, 118±39.8 μm/s^2; align, 269.3±37.8 μm/s^2; **(G)** contraction deformation distance – flat, 2.1±0.3 μm; random, 0.7±0.1 μm; align, 1.8±0.1 μm; and **(H)** relaxation deformation distance – flat, 2.0±0.2 μm; random, 0.6±0.1 μm; align, 1.8±0.2 μm. Data are presented as means ± standard deviation (SD). *P < 0.05, **P < 0.01, ****P < 0.0001; n = 3.

Supplementary Figure 3

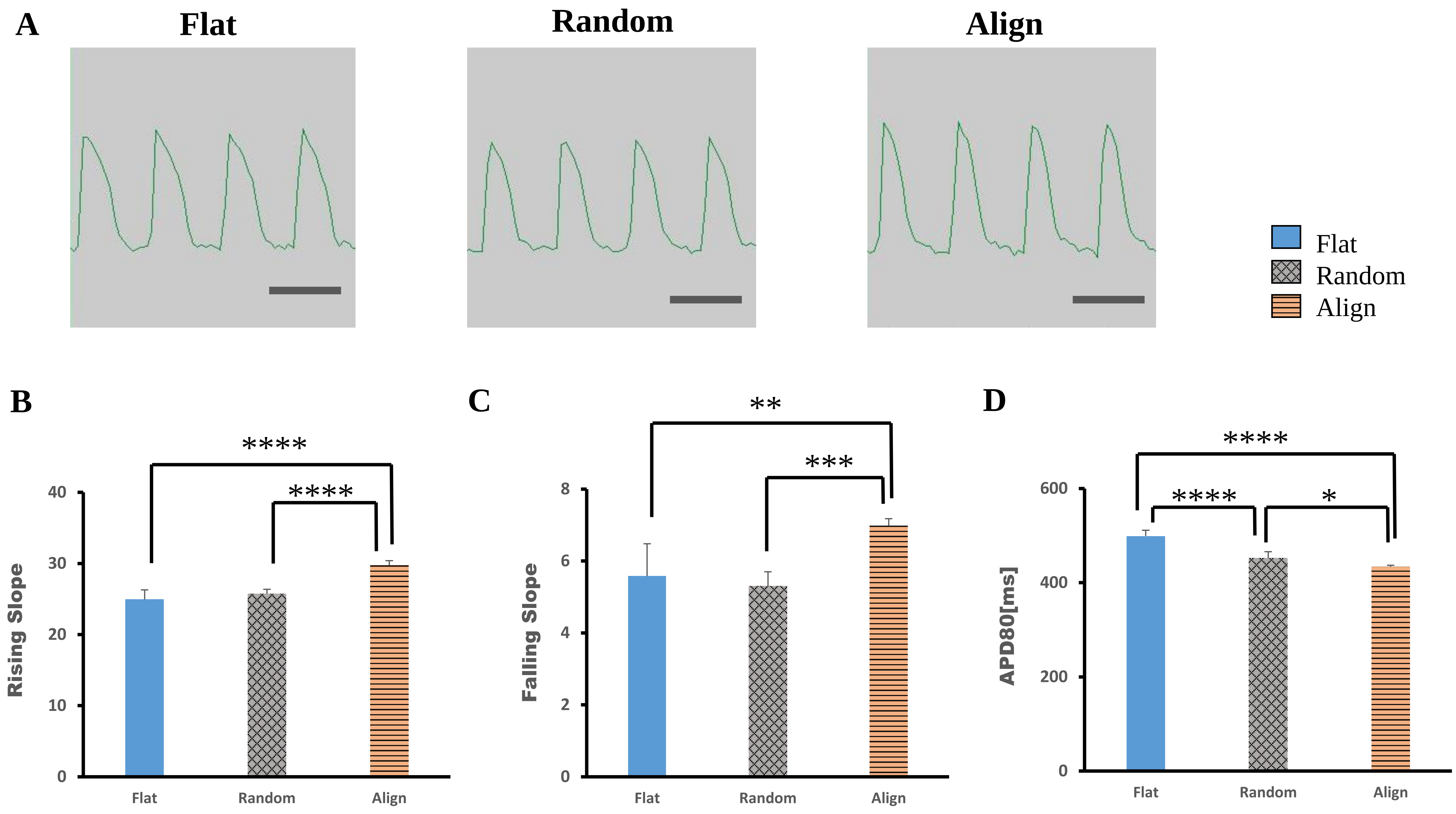

Supplementary Figure 3. (A-D) Action potential of 253G1 hiPS-CMs. (A) Action potential representative waveforms of hiPS-CMs on flat, random, and align patterns. Time scale bar: 1 s. Comparison of the action potential parameters; **(B)** rising slope – flat, 24.93±1.37; random, 25.71±0.66; align, 29.76±0.64; **(C)** falling slope – flat, 5.58±0.90; random, 5.30±0.40; align, 6.98±0.20; and **(D)** action potential duration at 80% repolarization (APD80) – flat, 499.28±12.18 ms; random, 452.41±13.24 ms; align 433.87±3.37 ms. Data are presented as means ± standard deviation (SD). *P < 0.05, **P < 0.01, ***P < 0.001, ****P < 0.0001; n = 3.

Supplementary Figure 4

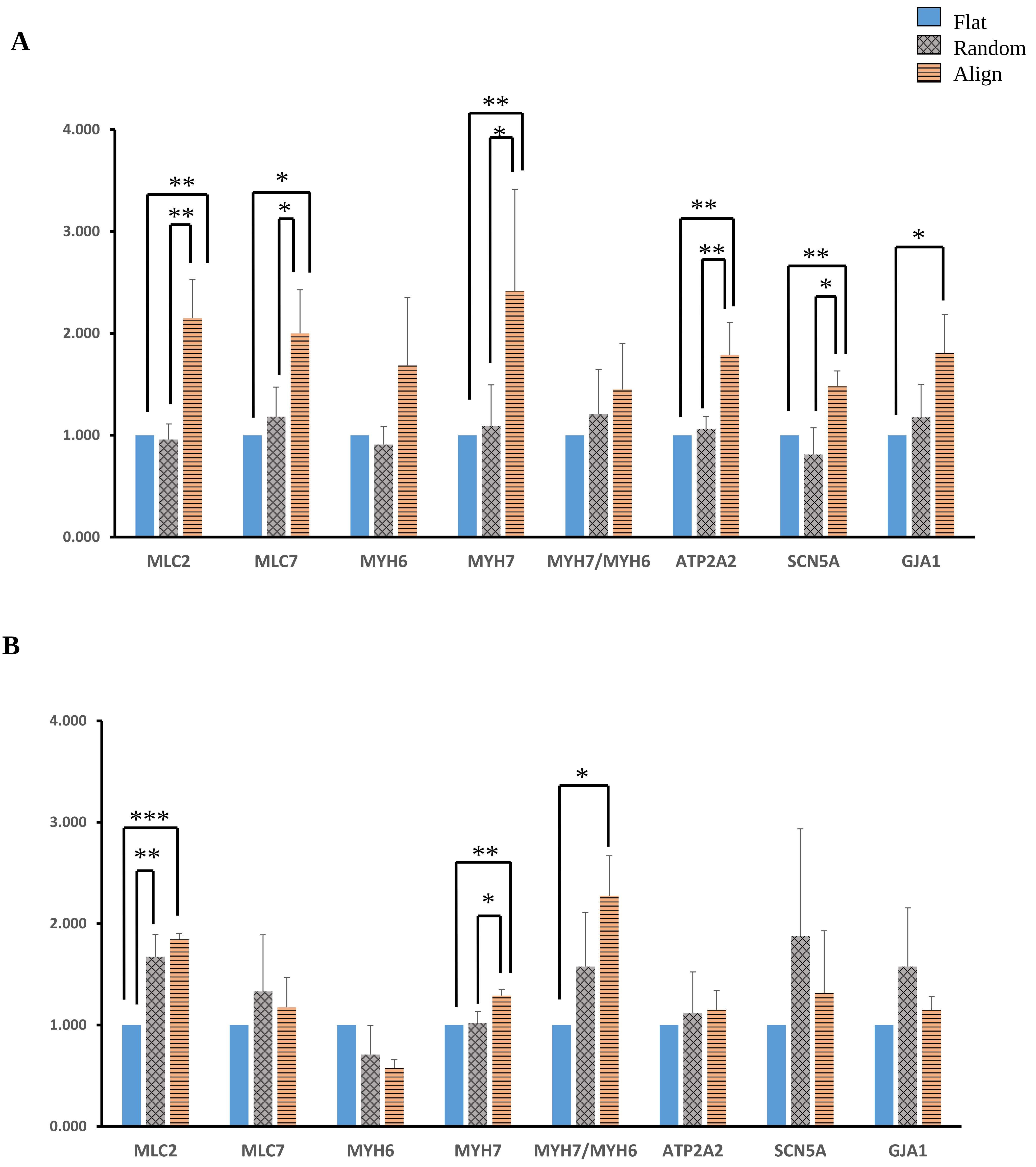

Supplementary Figure 4. (A) Cardiac gene expression on 253G1 hiPS-CMs on the flat, random, and align patterns. Comparison of the expression of cardiac structural genes, such as myosin light chain 2 (MYL2), myosin light chain 7 (MYL7), α-myosin heavy chain (MYH6), β-myosin heavy chain (MYH7); gap junction connexin-43 (GJA1); calcium handling-associated genes sarco/endoplasmic reticulum Ca2+-ATPase (SERCA2/ATP2A2); and conduction-related genes such as fast sodium (Na+) ion channel (SCN5A) on 253G1 hiPS-CMs on the flat, random, and align patterns. The expression of each gene was analyzed using quantitative reverse transcription-polymerase chain reaction (qRT-PCR) and normalized to 18S rDNA expression. *P < 0.05, **P < 0.01; n = 3. **(B)** Cardiac gene expression on 253G1 hiPS-CMs after replating on non-scaffold (flat bottom) pattern. Each gene expression was normalized to 18S rDNA expression. *P < 0.05, **P < 0.01, ***P < 0.001; n = 3.

Supplementary Figure 5

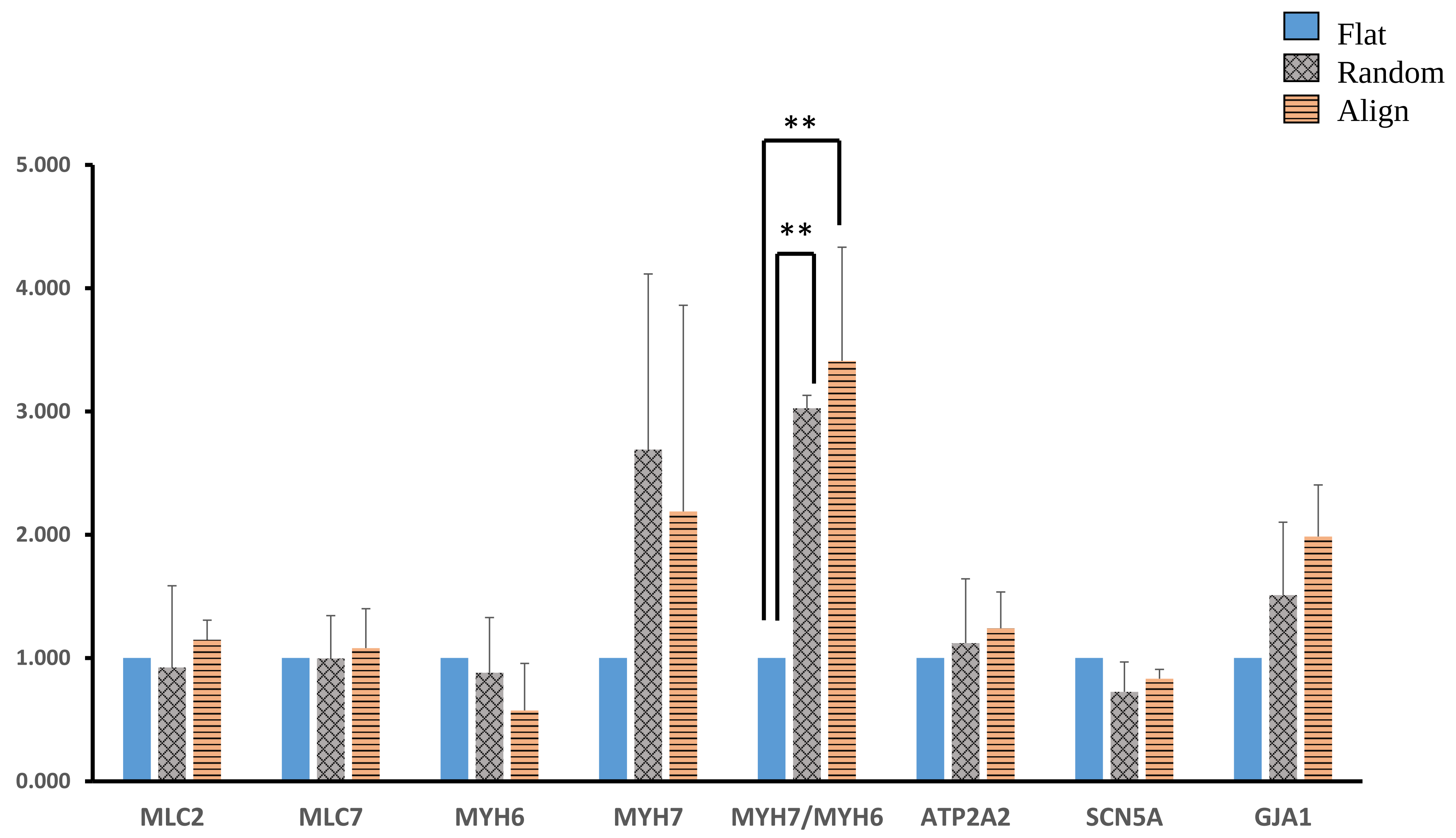

Supplementary Figure 5. Cardiac gene expression on 201B7 hiPS-CMs after replating on non-scaffold (flat bottom) patterns for 2 weeks. Comparison of the expression of cardiac structural genes, such as MYL2, MYL7, MYH6, MYH7; gap junction connexin-43 (GJA1), calcium handling-related genes SERCA2/ATP2A2, and conduction-related genes, such as SCN5A in hiPS-CMs pre-cultured on flat, random, and align patterns. Each gene expression level was normalized to 18S rDNA expression. **P < 0.01; n = 3.

Supplementary Figure 6

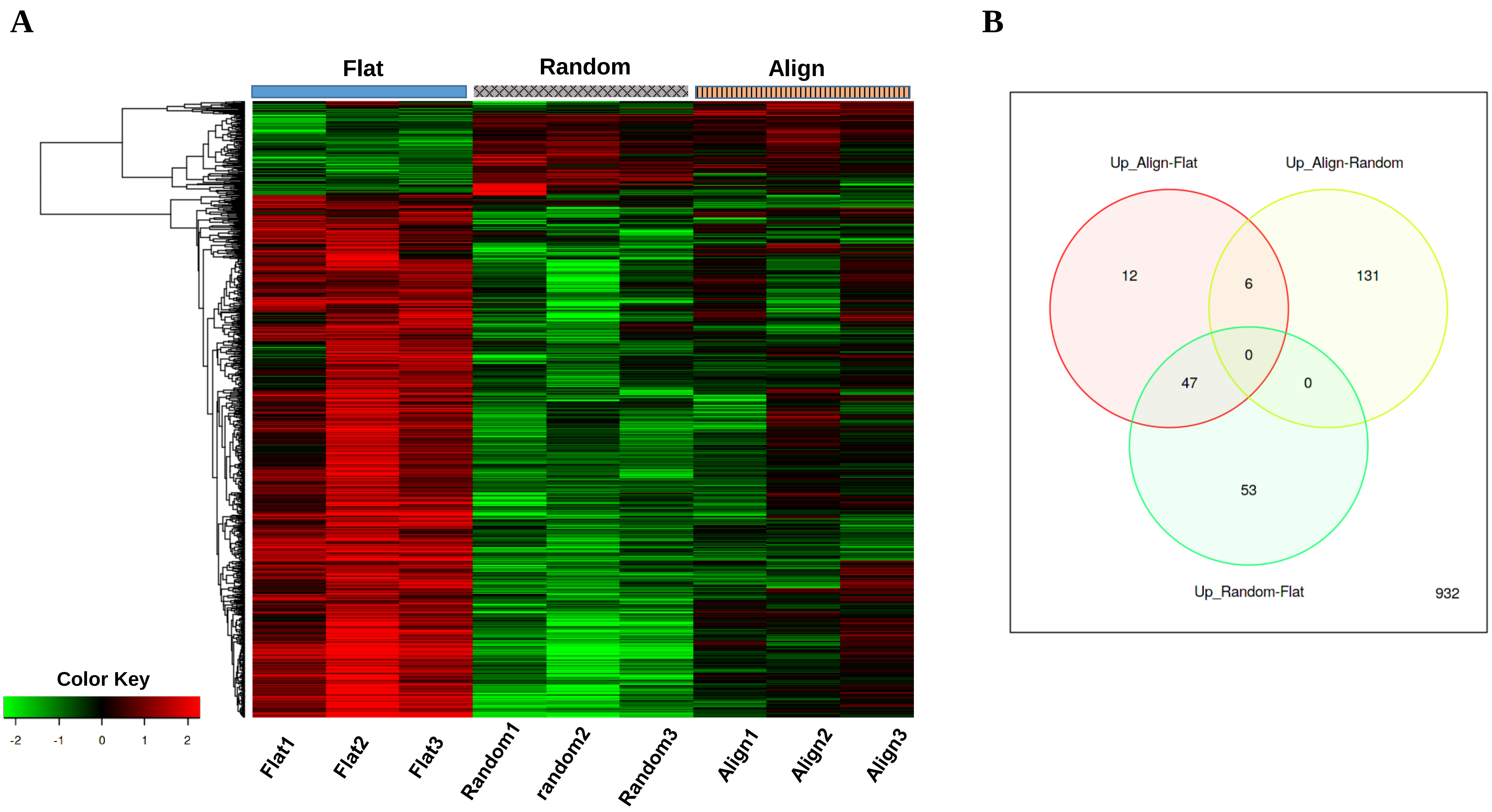

Supplementary Figure 6. (A) Heatmap showing the expression levels of differentially expressed genes in the flat, random, and align patterns. False discovery rate (FDR)<0.05, Cut-off Z score: $-2 < z < 2$; n = 3. **(B)** Venn diagram showing overlap of the upregulated genes in the align pattern compared to the flat and random groups, and overlap with upregulated genes in the random group compared to the flat group. FDR<0.1 and fold-change > 2; n = 3.
